# The Physiological and Evolutionary Ecology of Sperm Thermal Performance

**DOI:** 10.3389/fphys.2022.754830

**Published:** 2022-03-24

**Authors:** Wayne Wen-Yeu Wang, Alex R. Gunderson

**Affiliations:** Department of Ecology and Evolutionary Biology, Tulane University, New Orleans, LA, United States

**Keywords:** climate change, fertility, heat stress, postcopulatory selection, spermatogenesis, spermatozoa, thermal adaptation, thermal plasticity

## Abstract

Ongoing anthropogenic climate change has increased attention on the ecological and evolutionary consequences of thermal variation. Most research in this field has focused on the physiology and behavior of diploid whole organisms. The thermal performance of haploid gamete stages directly tied to reproductive success has received comparatively little attention, especially in the context of the evolutionary ecology of wild (i.e., not domesticated) organisms. Here, we review evidence for the effects of temperature on sperm phenotypes, emphasizing data from wild organisms whenever possible. We find that temperature effects on sperm are pervasive, and that above normal temperatures in particular are detrimental. That said, there is evidence that sperm traits can evolve adaptively in response to temperature change, and that adaptive phenotypic plasticity in sperm traits is also possible. We place results in the context of thermal performance curves, and encourage this framework to be used as a guide for experimental design to maximize ecological relevance as well as the comparability of results across studies. We also highlight gaps in our understanding of sperm thermal performance that require attention to more fully understand thermal adaptation and the consequences of global change.

## Introduction

Global climate change is expected to have broad effects on the biodiversity of Earth, and our knowledge about how organisms will respond to ongoing environmental disturbance still needs considerable improvement (IPCC, 2014). Many studies have measured the thermal performance and lethal heat thresholds of species to examine the ecological and evolutionary impact of rising global temperature, with the ultimate goal of making inferences about how population processes will be affected (summarized in Seebacher et al., 2014; Gunderson and Stillman, 2015; Sinclair et al., 2016; Pinsky et al., 2019; Buckley and Kingsolver, 2021). However, these studies tend to focus on diploid adult or sub-adult individuals. Far fewer studies have focused on how sublethal temperatures impact reproductive cells, even though they have direct influence on fertility and fitness. For example, a recent study on 43 *Drosophila* species showed that male sterility temperatures better predict species global distributions than the upper lethal temperatures of adults (Parratt et al., 2021). This result emphasizes the significant role male gametes can play in shaping ecological pattern and process, and their likely importance in dictating how biodiversity will react to global warming.

Sperm are one of the only cell types that are released into a foreign environment after maturing, traveling through and interacting with the external physical environment and/or the female reproductive tract (Pitnick et al., 2009, 2020). During the life cycle of sperm, from development in the testis to egg fertilization, sperm may experience a wide range of environmental conditions including variation in temperature, pH, ionic state, and viscosity (Reinhardt et al., 2015). Here we focus on temperature, one of the most significant environmental factors relevant to environmental adaptation and climate change that could impact sperm traits and their ability to fertilize. In general, studies of temperature-dependent sperm performance are concentrated on model organisms used for medical or domestic breeding purposes, and therefore lack ecological and evolutionary context (Reinhardt et al., 2015; Lüpold and Pitnick, 2018; Walsh et al., 2019). From these studies, we know the impact of thermal stress on sperm traits can include morphological and behavioral changes, decreased sperm count and longevity, and increased DNA damage (Cameron and Blackshaw, 1980; Foldesy and Bedford, 1982; Flowers, 1997, 2015; Blanckenhorn and Hellriegel, 2002; Reinhardt et al., 2015; Parrish et al., 2017; Peña et al., 2021). Therefore, as thermal niches of populations diverge, we expect the temperature-dependence of sperm performance to undergo adaptive evolutionary change in concert with other aspects of organismal performance. However, considerably less is known about ecologically relevant sperm thermal biology in wild populations. This is slowly changing, however, and exciting results are beginning to emerge.

**FIGURE 1:**
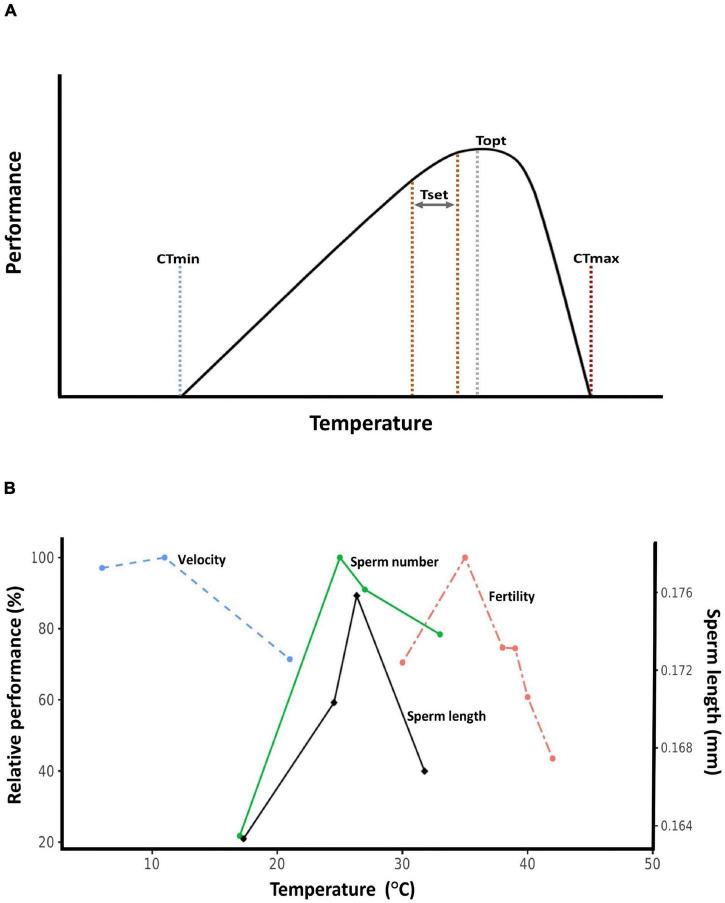
Thermal performance curves. **(A)** Hypothetical thermal performance curve. CTmin: critical thermal minimum, the cold tolerance limit. CTmax: critical thermal maximum, the heat tolerance limit. Set point range (Tset): also known as the preferred temperature range, this is the target range of body temperatures ectothermic organisms seek out in a thermally variable environment. Topt: thermal optimum, the temperature at which performance is maximized. **(B)** Relative thermal performance (maximum value set to 100%) of ejaculated sperm velocity (Atlantic cod, *Gadus morhua*, Purchase et al., 2010), developmental sperm length (bruchid beetle, *Callosobruchus maculatus*, Vasudeva et al., 2014), sperm number (bruchid beetle, *C. maculatus*, Vasudeva et al., 2014), and male fertility (red flour beetle, *Tribolium castaneum*, Sales et al., 2018).

Here, we review empirical evidence for how temperature, and especially high temperature, affect sperm traits. We focus on data from wild animal populations whenever possible, but include data from plants and domesticated organisms when appropriate. We also attempt to place data within the “thermal performance curve” framework (Huey and Kingsolver, 1989). Thermal performance curves describe how rates of biological processes change with temperature, and are applicable to everything from enzymatic catalysis to population growth rates (Angilletta, 2009; Somero et al., 2017). The crux of thermal performance curves is that they are unimodal and asymmetrical: performance peaks at a temperature closer to the heat tolerance limit than to the cold tolerance limit (Figure 1A). This means that organisms tend to live at body temperatures close to their heat thresholds, making them vulnerable to warming (Deutsch et al., 2008; Huey et al., 2009). Data on sperm thermal traits are rarely discussed within the performance curve context (Figure 1B), but we believe doing so is beneficial for understanding the evolutionary ecology of sperm traits. Throughout, we attempt to synthesize emerging patterns from the available data, and also highlight gaps in our understanding that require greater attention.

Our review includes morphological and performance aspects of sperm phenotypes. We apply the term “performance” broadly, including any aspect of sperm-related phenotypes (number, percent motility, velocity, and fertilization success) that is not explicitly morphological.

## How Temperature Affects Sperm Traits

**FIGURE 2:**
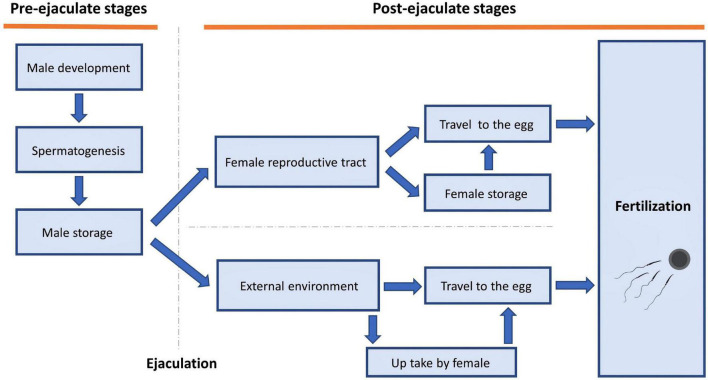
Stages at which temperature could impact sperm traits.

Temperature can affect sperm traits in many ways, both pre- and post-ejaculation (Figure 2). Below, we step through examples of how temperature can affect sperm traits at different stages of the sperm life cycle.

### Pre-ejaculate Thermal Effects

Male developmental temperature plays an important role in testis development (Blanckenhorn and Henseler, 2005; Sales et al., 2021), which could subsequently affect sperm traits (Blanckenhorn and Hellriegel, 2002; Vasudeva et al., 2014; Sales et al., 2021; but see Iglesias-Carrasco et al., 2020). In yellow dung flies (*Scathophaga stercoraria*), individuals produced the longest sperm after undergoing development at either intermediate or high temperature (Blanckenhorn and Hellriegel, 2002). In the pseudoscorpion (*Cordylochernes scorpiodes*), fewer sperm were produced and fertility declined when reared at higher temperature (Zeh et al., 2012). The effects of heat stress can also vary across development stage, and male fertility may be more sensitive to heat during testicular development relative to earlier developmental stages. For example, red flour beetles (*Tribolium castaneum*) exposed to heat shock during the pupa and immature adult stage when testis are developing had to decreased testis volume, sperm number, sperm viability, and fertility; conversely, heat shock during an earlier larval stage had no effect on reproductive traits (Saxena et al., 1992; Sales et al., 2021).

Studies capable of isolating the effect of temperature on sperm through effects on testis development are rare (Sales et al., 2021). This is because many studies maintain males at their developmental temperature through sexual maturity, at which point sperm analysis occurs. As a result, it is not possible to disentangle effects that emerge due to testis development from direct effects on spermatogenesis or stored sperm. Regardless of the specific mechanism, studies often find that pre-ejaculate temperatures affect sperm traits. For instance, fewer and shorter sperm were produced by bruchid beetles (*Callosobruchus maculatus*) reared until sperm analysis at the highest and lowest experimental temperatures (Vasudeva et al., 2014), while field crickets (*Gryllus bimaculatus*) produced the most sperm when they developed at high temperature (Gasparini et al., 2018). In Trinidadian guppies (*Poecilia reticulata*), sperm length and swimming velocity were lowest for males reared until sperm analysis in the warmest treatment groups (Breckels and Neff, 2013, 2014).

Once males have reached sexual maturity, the temperature they experience can greatly affect sperm. For example, adult boar (*Sus scrofa domestica*) exposed to high temperature produce sperm with higher levels of DNA damage (e.g., decreased DNA integrity), more morphological abnormalities, and lower motility than sperm from boar housed under normal or cooler thermal conditions (Cameron and Blackshaw, 1980; Flowers, 1997, 2015; Parrish et al., 2017; Peña et al., 2021). Similar effects occur in bull (*Bos taurus*; Cheng et al., 2016; Morrell, 2020), ram (*Ovis aries*; Rathore, 1970; Küçük and Aksoy, 2020), zebra finch (*Taeniopygia guttata*; Hurley et al., 2018), and chicken (*Gallus gallus domesticus*; Karaca et al., 2002). Such studies in wild endotherms are rare. To the best of our knowledge, the only applicable data come from the African lion (*Panthera leo*). Male lions with darker and thicker manes have higher body and testicular temperature and produce more morphologically abnormal sperm than lions with blonde manes (West and Packer, 2002).

Studies that increase testicular temperature specifically have also found large impacts on ejaculated sperm traits. For example, raising the testis temperature of laboratory rats to core body temperature by sealing the testis into the abdomen leads to a significant decrease in sperm motility and male fertility (Foldesy and Bedford, 1982). Negative effects are also observed in experiments in which the scrotum is insulated to increase temperature. For example, scrotal insulation studies in livestock and humans have found decreased sperm concentration, motility, and viability, as well as increased morphological abnormalities and DNA damage (Vogler et al., 1993; Abdelhamid et al., 2019; Garcia-Oliveros et al., 2020; Shahat et al., 2020).

The above examples focus on endotherms, but heat exposure after maturity also has a dramatic effect on ectotherm sperm traits. For instance, male red flour beetles (*T. castaneum*) produced 75% less sperm and had decreased sperm viability after exposure to a simulated heatwave (Sales et al., 2018, 2021; Vasudeva et al., 2021). Similar effects on sperm number and motility occur in field crickets (*G. bimaculatus*; Gasparini et al., 2018). In Brown trout (*Salmo trutta*), males exposed to warmer temperature showed no change in the proportion of motile cells, but produced significantly slower sperm (Fenkes et al., 2017). In some organisms, temperatures do not need to be much higher than “normal” for sperm development to be affected. For example, in the coral (*Acropora digitifera*), adults produced significantly fewer sperm during spawning after experiencing only a 2°C elevation of water temperature above ambient for 1 month (Paxton et al., 2015).

These effects can have subsequent repercussions for reproduction. For example, in the flesh fly (*Sarcophaga crassipalpis*), males became completely infertile after heat shock, and very few sperm were found in females mated to heat shocked males (Rinehart et al., 2000). Similar findings have been shown in red flour beetles (*T. castaneum*; Sales et al., 2018, 2021) and *C. elegans* (Aprison and Ruvinsky, 2014; Poullet et al., 2015). Trans-generational effects of temperature exposure have also been found in field crickets (*G. bimaculatus*), where adult males that experienced a warm treatment produced lower quality sperm and offspring with lower survival rate compared with cool treated males (Gasparini et al., 2018).

Though lacking information on specific sperm traits, several additional studies have found that male fertility is affected by temperatures they experience after maturity. For example, in many fruits flies (*Drosophila*), heat stressed males produce significantly fewer offspring, and the effect increases with the duration of heat exposure (David et al., 2005). Although these males were usually able to regain fertility through time, many were permanently sterile after exposure to prolonged or more extreme heat stress (Jørgensen et al., 2006; Sutter et al., 2019; van Heerwaarden and Sgrò, 2021). Similar effects of heat on male fertility have also been shown in Diamondback Moths (*Plutella xylostella*; Zhang et al., 2013). Male sterility after heat exposure was likely caused by a change in sperm traits, since normal mating behavior was observed in these studies (Jørgensen et al., 2006; Zhang et al., 2013; Sutter et al., 2019). In response to heat-induced male sterility, females may engage in mating with more males to maintain their own fecundity. By accumulating stored sperm through multiple mating, females can potentially restore their fecundity to normal levels (*Drosophila pseudoobscura*; Sutter et al., 2019; red flour beetles, *T. castaneum*; Vasudeva et al., 2021).

It is worth noting that male reproductive traits seem to be more sensitive to heat than female reproductive traits, based on studies in which the effects of temperature can be inferred for both sexes. In field crickets and flour beetles, heat treatments that affected males sperm traits did not affect female egg number (Paxton et al., 2015) or reproductive output (Sales et al., 2018, 2021). Furthermore, in the nematodes *C. elegans* and *C. briggsae*, infertility after heat stress is mainly due to defective sperm function (Prasad et al., 2011; Aprison and Ruvinsky, 2014; Poullet et al., 2015; but see Janowitz and Fischer, 2011). More work is required to determine the generality of these findings.

Overall, the evidence indicates that high temperatures can have major consequences for sperm quality and performance. Indeed, this is highlighted by the many thermoregulatory mechanisms that organisms have evolved to protect sperm from heat. For example, most mammals maintain testis temperatures 1–6°C below core body temperature, facilitated by morphological and physiological specialization in the testicles (Cowles, 1958; Rommel et al., 1992; Pabst et al., 1995; Thundathil et al., 2012).

In ectotherms, thermoregulation is primarily behavioral. Taxa have a target range of body temperatures (the preferred temperature range, Tpref) that they seek out within their habitats. Tpref is expected to evolve in concert with other physiological adaptations to temperature such that animals prefer body temperatures that confer high physiological performance (Huey et al., 1999; Angilletta et al., 2006). Classic early papers in reptiles contended that Tpref evolution is tied to the thermal dependence of testis health in males (reviewed in Dawson, 1975). For example, exposing mature males to temperatures 1–2°C above Tpref led to testicular tissue damage in the lizards *Urosaurus ornatus*, *Sceloporus virgatus*, and *S. graciosus* (Licht, 1965). Similar results were found using cultured testicular tissue from the lizards *Anolis carolinensis* and *Uma scoparia* (Licht and Basu, 1967). These observations highlight that thermoregulation plays a central role in the ecology and evolution of temperature-dependent sperm performance and warrants further attention within that context.

### Post-ejaculate Thermal Effect: Direct Effect on Mature Sperm Cells

After ejaculation, the temperature of the environment has a direct effect on sperm performance (reviewed in Alavi and Cosson, 2005; Revathy and Benno Pereira, 2016). For example, the sperm of external fertilizers may experience a wide range of ambient temperatures before reaching the eggs (Chirgwin et al., 2019; Walsh et al., 2019). Several studies have shown increased sperm velocity and/or flagellar beat frequency at higher water temperatures (Kupriyanova and Havenhand, 2005; Lahnsteiner and Mansour, 2012; Fenkes et al., 2017; Iglesias-Carrasco et al., 2020). That said, studies that include a wide range of test temperatures usually find that velocity is maximized at intermediate temperatures, consistent with a thermal performance curve (Alavi and Cosson, 2005; Purchase et al., 2010; Lahnsteiner and Mansour, 2012).

In contrast, high temperature tends to decrease sperm longevity, likely due to increased metabolic rates that rapidly deplete finite energy reserves (Schlenk and Kahmann, 1938; Alavi and Cosson, 2005; Bombardelli et al., 2013). For example, southern hake (*Merluccius australis*) sperm longevity was greatest when incubated at 4°C and decreased progressively with incubation at 10 and 15°C (Effer et al., 2013; but see Kekäläinen et al., 2018; Lymbery et al., 2020). The consequences of variation in environmental temperature for sperm performance can translate to fertilization success. For example, fertilization capacity decreases significantly if ejaculated sperm are exposed to heat in multiple sea urchin species (Rahman et al., 2009).

Exposing ejaculated sperm to heat may also have trans-generational effects. In European whitefish (*Coregonus lavaretus*), offspring sired by heat exposed sperm were smaller and had poorer swimming ability (Kekäläinen et al., 2018). Conversely, positive effects have been found in mussels (*Mytilus galloprovincialis*), where offspring survival rate was higher when sired by heat exposed sperm (Lymbery et al., 2021). These trans-generational effects may be caused by epigenetic changes or selection on sperm haplotypes (Lymbery et al., 2020, 2021). However, the underlying mechanisms are far from clear and need further testing.

Fertilization success changes with water temperature in external fertilizers, although it is difficult to determine the relative contribution of sperm and egg to this pattern. For instance, in tube worms (*Galeolaria caespitosa*), the thermal performance curve for fertilization rate shows maximum fertilization success at 21°C, which is the typical water temperature in their native habitat during summer (Kupriyanova and Havenhand, 2005). In a different study, fertilization rates of three-spined sticklebacks (*Gasterosteus aculeatus*) were lowest when gametes were exposed to the higher of two temperatures (Mehlis and Bakker, 2014).

Temperature also affects sperm performance in internal fertilizers, and high temperature in particular tends to result in negative effects (Monterroso et al., 1995; Chandolia et al., 1999). For example, the motility of boar (*S. scrofa domestica*) sperm decreased by 32% when incubated at 40 versus 38.5°C, the latter temperature closer to body temperature (Calle-Guisado et al., 2017; Gong et al., 2017). In New Zealand white rabbits (*Oryctolagus cuniculus*), sperm motility and metabolic activity decreased significantly after incubation above body temperature for 3 h (Sabés-Alsina et al., 2016). Available evidence is broadly consistent in ectotherms (Sales et al., 2018). For example, decreased motility and velocity of ejaculated sperm at high temperature has been observed in lizards (Rossi et al., 2021) and snakes (Tourmente et al., 2011).

An important consideration when analyzing the performance of ejaculated sperm in internal fertilizers is the influence of the female reproductive tract. The female reproductive system interacts with sperm and influences sperm performance and fertilization success by dictating the physical and chemical environment that sperm experience (Pitnick et al., 2009; Rosengrave et al., 2009; Sasanami et al., 2013; Lüpold and Pitnick, 2018; Rossi et al., 2021). Though rarely studied, available evidence suggests this interaction is important for sperm thermal performance. For example, oviductal fluid extracted from females reduced the negative effect of high incubation temperature on sperm in spiny lava lizards (*Tropidurus spinulosus*). Specifically, sperm incubated at high temperature with oviductal fluid had significantly higher motility and velocity compared to those without oviductal fluid (Rossi et al., 2021).

That said, protection provided by the female likely only goes so far. Ejaculated sperm stored within the female reproductive system can still be effected by temperature. In the spiny lizard example, the performance of sperm incubated with oviductal fluid still decreased at high temperature (Rossi et al., 2021). Indirect evidence for limits on female capacity to buffer sperm from high temperature damage is found in a study of the red flour beetle (*T. castaneum*). They found that heat exposure dramatically reduced female fertility; however, that was only true if females were mated beforehand. Females mated after heat exposure did not experience reduced fertility. Though not conclusive, this pattern is consistent with high temperature negatively affecting sperm after insemination (Sales et al., 2018; Vasudeva et al., 2021). If sperm are influenced by female body temperature after insemination, female thermoregulation will also play a key role in the ecology and evolution of sperm performance, especially in species with long-term female storage.

## Is Phenotypic Plasticity in Sperm Traits Adaptive or Not?

Phenotypic plasticity refers to the ability of a genotype to express different phenotypes based on environmental conditions (Pigliucci, 2001). If plasticity occurs in a manner that improves performance, it is considered adaptive (Huey et al., 1999; Wilson and Franklin, 2002). Adaptive phenotypic plasticity is thought to be an important mechanism that can help organisms cope with temperature fluctuations (Somero, 2010; Seebacher et al., 2014; Gunderson and Stillman, 2015).

Explicit tests of adaptive phenotypic plasticity in sperm traits are rare, but there is compelling evidence that it can occur. Perhaps the best evidence comes from a study of red flour beetles (*T. castaneum*). Male beetles were acclimated to either standard or warm conditions, and were then mated to standard-reared females under the male acclimation temperature. After mating, females were move into either standard or warm conditions to reproduce. The results showed that females were able to produce significantly more offspring when their post-mating temperature matched male acclimation temperature. In other words, sperm performance was greatest under temperatures to which males were acclimated (Vasudeva et al., 2019). However, adaptive sperm thermal plasticity has not been supported in some other studies. In brown trout (*S. trutta*) and European whitefish (*C. lavaretus*), adult males acclimated to warm temperatures did not produce sperm with improved thermal tolerance or relative motility under warm temperatures (Fenkes et al., 2017; Kekäläinen et al., 2018). Similarly, no adaptive plasticity has been found in response to either rearing or adult acclimation temperature in mosquitofish (*G. holbrooki*; Adriaenssens et al., 2012; Iglesias-Carrasco et al., 2020).

Another approach to inferring adaptive plasticity is to compare the direction of plastic phenotypic change to the direction of evolutionary phenotypic change under the same conditions (Ghalambor et al., 2015). Interestingly, two studies that have compared evolutionary and plastic responses of sperm traits to temperature within the same taxa found that they occur in opposite directions: decreased sperm length was the plastic response to heat, while increased sperm length was the evolutionary response to heat (Breckels and Neff, 2013; Vasudeva et al., 2019). This suggests that some plasticity in temperature-dependent sperm traits could be maladaptive, and evolution acts to overcome that maladaptive response (Ghalambor et al., 2015).

Phenotypic plasticity is typically underlain by changes in gene regulation, and temperature-dependent transcript plasticity has been shown when individuals or their ejaculated sperm experience heat stress. For example, male domesticated bulls (*B. taurus*) produce sperm with an increased abundance of transcripts for heat shock protein genes *hsp60* and *hsp70* after exposure to heat (Cheng et al., 2016). In contrast, lower levels of *hsp90* but not *hsp70* mRNA were found in ejaculated sperm of mussels (*M. galloprovincialis*) after ejaculated sperm were exposed heat, though no phenotypic changes in sperm motility were observed (Lymbery et al., 2020). Whether differences in transcript abundance were due to gene regulation, degradation of specific RNAs, and/or changes in translation remain unclear (Lymbery et al., 2020). Heat-induced changes in transcript profiles, particularly for *hsp* genes, could imply an adaptive response since these genes code for molecular chaperones that maintain cellular function under thermal stress and are important in sperm development and function (Huang et al., 2000; Dun et al., 2012; Meccariello et al., 2014). However, further studies will need to be done to establish any adaptive significance.

## Evidence of Evolutionary Diversification in Sperm Thermal Performance

From an evolutionary perspective, sperm traits are expected to be selected to maximize fertilization success under prevailing environmental conditions (Pitnick et al., 2009; Reinhardt et al., 2015). This view has been supported by studies on sperm morphology. For example, in many species, sperm length co-evolves with different aspects of female reproductive tract (Miller and Pitnick, 2002; Pitnick et al., 2009; Breckels and Neff, 2013). However, we know relatively little about adaptive divergence in sperm physiological traits with respect to abiotic environmental factors such as temperature (Reinhardt et al., 2015). From our review of the literature, there seems to be a correlation between adult thermal tolerance and the heat tolerance of sperm production and/or post-ejaculate performance. For example, several studies have compared sperm thermal performance between two or three species with divergent thermal niches and have found that performance at high temperature is greater in the warmer niche species (Rahman et al., 2009; Tourmente et al., 2011; Lahnsteiner and Mansour, 2012; Poullet et al., 2015; Revathy and Benno Pereira, 2016).

**FIGURE 3:**
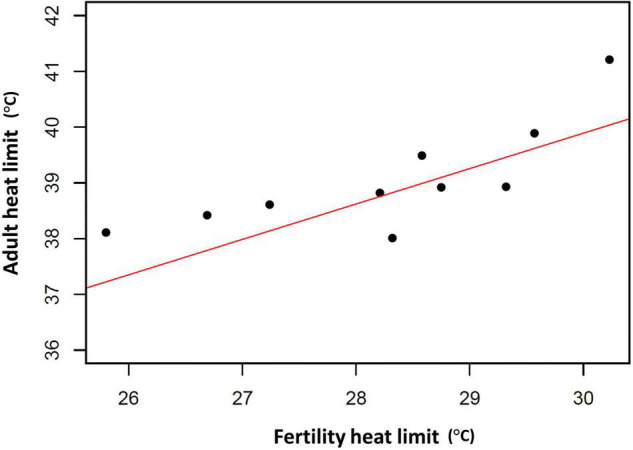
Association between adult critical thermal maximum (CTmax) and male fertility thermal limits (FTL50) among 10 *Drosophila* species. Red line represents the fitted phylogenetic generalized least squares (PGLS) model fitted using the R package nlme (*R*^2^ = 0.59, *t* = 5.59, *d.f.* = 10, *p* < 0.001). CTmax and FTL50 data from van Heerwaarden and Sgrò (2021). Phylogenetic information from Finet et al. (2021).

**FIGURE 4:**
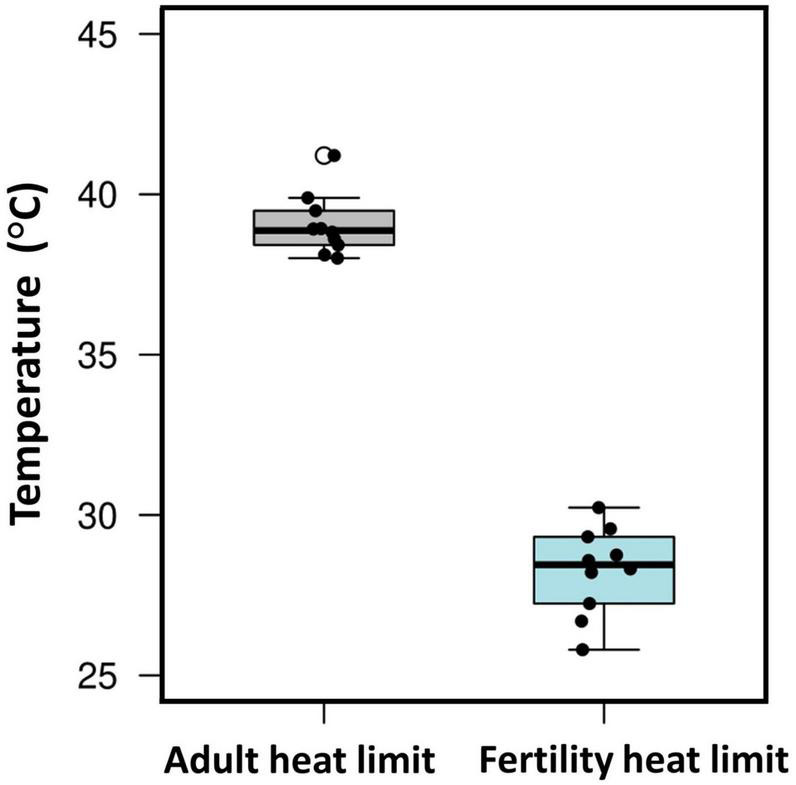
Male fertility is more sensitive to heat than adult thermal tolerance. Box plot showing male fertility thermal limits (FTLs) and critical thermal maximum (CTmax) from 10 *Drosophila* species (data from van Heerwaarden and Sgrò, 2021). Boxes include the interquartile range, the whiskers span the range of the data, and the thick bars indicate the median values. Each point refers to data for a single species.

Of course, evolutionary adaptation cannot be inferred from two-species studies (Garland and Adolph, 1994). More compelling evidence comes from studies of divergence in male thermal sterility thresholds. For example, male whole-organism heat tolerance, a proxy for thermal niche, highly corelates with male sterility temperature across many Drosophilinae fly species (Figure 3; David et al., 2005; Parratt et al., 2021; van Heerwaarden and Sgrò, 2021). In addition, male fertility limits were found to correlate strongly with geographic distribution (and thus thermal environment) across many *Drosophila* species, providing further evidence for adaptive divergence in temperature-dependent male reproductive traits (Parratt et al., 2021). Interestingly, in almost every species, male fertility thermal (heat) limits (FTL) are lower than the adult heat limits (Figure 4), indicating that male fertility is more sensitive to heat than the whole organism (David et al., 2005; Jørgensen et al., 2006; Parratt et al., 2021; van Heerwaarden and Sgrò, 2021).

At the population level, divergence in sperm morphological traits is commonly reported (Kuramoto, 1996; Pitnick et al., 2003; Hettyey and Roberts, 2006; Minoretti and Baur, 2006; Kustra et al., 2019). Data on population-level divergence in environment-dependent performance is much more limited. For example, Green et al. (2019) found evidence of adaptive sperm evolution in response to local salinity conditions in two invasive populations of round goby (*Neogobius melanostomus*). Sperm from the high salinity population had greater sperm motility under high salinity conditions than those from the low salinity population (Green et al., 2019). Few studies have tested for intraspecies divergence in sperm thermal performance, but examples do exist. Sperm from a low latitude (i.e., warmer climate) population of *Drosophila subobscura* were less affected by heat than those from a high latitude population (Porcelli et al., 2017). Similarly, intraspecific variation in the heat sensitivity of fertility is largely consistent with habitat temperature in *D. melanogaster* (Rohmer et al., 2004) and the nematodes *C. briggsae* and *C. tropicalis* (Prasad et al., 2011; Poullet et al., 2015).

For sperm traits to evolve under different thermal regimes, there must be genetic variation in the traits among individuals. Individual-level variation in sperm thermal performance has been reported in Atlantic cod (*Gadus morhua*), where a genotype by temperature interaction was shown for sperm swimming speed (Purchase et al., 2010). Similarly, individual variation in sperm trait thermal plasticity has been reported in domesticated animals that experience scrotal insulation (Vogler et al., 1991; Barth and Bowman, 1994). Individual variation in sperm performance opens the door for temperature-dependent sperm competition. Furthermore, if adult thermal traits are genetically linked to sperm thermal traits, selection *via* sperm competition could increase rates of thermal adaptation when habitat temperatures change. Although rarely tested in animals, temperature selection on the gamete level has been applied in plants. For example, pollen selection has been shown to increase chilling tolerance in chickpea (*Cicer arietinum*; Clarke et al., 2004) and heat tolerance in maize (*Zea mays* L.; Mohapatra et al., 2020).

Laboratory experimental evolution experiments have shown that sperm traits can rapidly evolve under divergent thermal regimes, though these experiments are rare. In the two studies we are aware of, evolution under warm temperature led to a decrease in sperm length over time in guppies (8 generations, Breckels and Neff, 2013) and in red flour beetles (over 50 generations, Vasudeva et al., 2019). Whether the evolutionary response of sperm length is adaptive, or is simply linked with other adaptive traits, remains unclear. In contrast, laboratory selection experiments in *Drosophila* found no evidence for evolution in male fertility thermal limits, and heat-induced infertility often leads to population extinction (van Heerwaarden and Sgrò, 2021).

## Implication Under Global Climate Change

The evidence reviewed above has obvious implications for the ability of species to tolerate anthropogenic global change. High temperatures can have strong negative effects on sperm performance. Importantly, these effects can develop at temperatures not far above normal body temperatures, and far below the lethal body temperatures of mature adults that are often used to model susceptibility to warming (Figure 4). The ability of sperm traits to rapidly evolve under high temperatures does provide some hope for adaptation to rapid climate change. So too does the potential for adaptive thermal plasticity in sperm traits. However, the relative dearth of studies that have investigated questions of thermal evolvability and adaptive plasticity in sperm traits leaves us with much to learn before we can draw any solid conclusions.

One approach that could greatly increase our understanding of how climate change and sperm thermal performance interact is greater integration of ecologically meaningful temperature data into experimental design. The conditions that organisms and sperm are experimentally exposed to should reflect what they experience in the wild, as well as what they may experience in the future (Helmuth et al., 2010; Sunday et al., 2014; Buckley et al., 2018). In many of the papers described above, habitat-specific environmental or, more importantly, body temperature data were not presented nor discussed with respect to experimental treatments. As a result, it is difficult to interpret the results in an ecological context. Studies that use unrealistically high treatment temperatures could be biased toward finding a negative effect of warming, pushing organisms above the thermal optimum of their thermal performance curve. Similarly, studies that use unrealistically cool temperatures could incorrectly predict that warming will increase sperm performance if they span temperatures below the thermal optimum of the thermal performance curve. Ideally, experiments will occur across a wide range of temperature such that a thermal performance curves can be estimated and ecologically relevant temperatures can be mapped onto that curve. Then, one can predict the effects of warming within populations and estimate relative vulnerability to warming among taxa (Huey et al., 2009).

Furthermore, treatments should subject experimental units (individuals or sperm) to ecologically relevant thermal fluctuations, not just constant temperatures. Constant-temperature treatments are common in physiological experiments (Gunderson et al., 2016), but exposure to more ecologically relevant fluctuating temperatures often elicit different physiological responses (Colinet et al., 2015; Marshall and Sinclair, 2015; Marshall et al., 2021). Only when ecologically relevant thermal data are more consistently incorporated into studies of sperm thermal physiology will we be able to determine how close wild organisms are to experiencing sperm performance detriments, how great performance detriments may be when temperatures change, and how the effects of warming may differ geographically and taxonomically.

## Conclusion

The weight of evidence makes it clear that temperature has a profound effect on sperm traits. These effects can manifest at many points in the life cycle of sperm and the tissues that produce and support them. Given the thermal sensitivity of sperm combined with the fact that they are so directly tied to reproductive fitness, we expect sperm traits to be under intense selection as species adapt to new thermal environments. Studies on the evolution of temperature-dependent sperm traits are relatively rare, but the data available suggest that divergence is common and may often be adaptive. Furthermore, experimental evolution experiments demonstrate that sperm traits can evolve rapidly when thermal conditions change.

Most studies of temperature-dependent sperm performance measured traits at only two or three temperatures, making inferences about thermal performance curve difficult or impossible. However, studies that applied a wide range of temperature treatments often found the classic thermal performance curve response for traits related to both morphology and performance (Figure 1B). Moving forward, we recommend that the thermal performance curve framework be used as a guide to design studies of thermal effects on sperm traits. Doing so will allow us to better understand the full spectrum of thermal effects on sperm and elucidate the ecological and evolutionary consequences of sperm phenotypes, as is the case for whole-organism thermal performance (Sinclair et al., 2016). In addition, application of the thermal performance curve perspective may help resolve inconsistencies observed among studies. For example, whether a study finds that warming increases, decreases, or has no effect on a trait of interest will depend on whether the treatment temperatures chosen fall on the rising (left), falling (right), or plateau section of the traits thermal performance curve. Ultimately, greater integration of sperm thermal responses to a wide range of ecologically relevant temperatures will be crucial to predict the effects of global warming on sperm and the subsequent consequences for reproduction and population processes.

## Author Contributions

WW and AG conceived the project and wrote the manuscript. Both authors contributed to the article and approved the submitted version.

## Conflict of Interest

The authors declare that the research was conducted in the absence of any commercial or financial relationships that could be construed as a potential conflict of interest.

## Publisher’s Note

All claims expressed in this article are solely those of the authors and do not necessarily represent those of their affiliated organizations, or those of the publisher, the editors and the reviewers. Any product that may be evaluated in this article, or claim that may be made by its manufacturer, is not guaranteed or endorsed by the publisher.
